# Supplementary material for: Substantial near-infrared radiation-driven photosynthesis of chlorophyll f-containing cyanobacteria in a natural habitat
Source: eLife. 2020 Jan 21;9:e50871. doi: 10.7554/eLife.50871 (PMC6974357; doi:10.7554/eLife.50871)
Supplement: Supplementary file 1. [file elife-50871-supp1.docx]

Overview of cyanobacterial strains and enrichments reported to contain Chl *f*
Listed in chronological order of first publication year.

| **Species / strain** | **Habitat / Strain origin** | **Growth form studied** | **Reference** |
| --- | --- | --- | --- |
| *Halomicronema hongdechloris* | Hypersaline, stromatolites, Shark Bay Western Australia | Filamentous, benthic (stromatolite) | *Chen et al., 2010; 2012* |
| *Aphanocapsa*-like, Strain KC1 | Freshwater, Lake Biwa, Japan | Unicellular, planktonic (algal bloom) | *Akutsu et al., 2011* |
| *Leptolyngbya* sp., Strain JSC-1 | LaDuke Hot Springs, USA | Filamentous, benthic (microbial mat) | *Gan et al., 2014* |
| *Chlorogloeopsis fritschii*, Strain PCC6912 | Rice paddy soil, India | Unicellular, planktonic | *Airs et al., 2014* |
| *Synechococcus* sp.  Strain PCC 7335 | Intertidal, Mexico | Unicellular, benthic  (biofilm) | *Gan et al., 2015* |
| *Chroococcidiopsis thermalis*, Strain PCC 7203 | Soil, Germany | Unicellular, benthic  (soil) | *Gan et al., 2015;* *Nürnberg et al., 2018* |
| *Calothrix* sp. Strain, PCC 7507 | Sphagnum bog, Switzerland | Filamentous, benthic | *Gan et al., 2015* |
| *Fisherella thermalis*,  Strain PCC 7521 | Hot spring, Yellowstone National Park | Filamentous, benthic  (microbial mat) | *Gan et al., 2015* |
| *Chlorogloeopsis* sp. Strain, PCC 9212. | Thermal spring, Spain | Unicellular, benthic  (microbial mat) | *Gan et al., 2015* |
| *Aphanocapsa*-like, KC1-related enrichment | Freshwater, cavernous, Jenolean Caves, Australia | Unicellular, benthic (epilithic biofilm) | *Behrendt et al., 2015* |
| *Chroococcidiopsis* spp., enrichment | Intertidal, beachrock, Heron Island, GBR, Australia | Unicellular, benthic (endolithic biofilm) | *Trampe and Kühl, 2016* |
| *Leptolyngbya* spp.  Strains Nb3F1, Nb3F2 | Nakabusa hot spring, Japan | Filamentous, benthic  (microbial mat) | *Ohkubo and Miyashita, 2017* |
| *Aphanocapsa sp.* Strain CCNUW3, | Freshwater macrophytes in rain forest ponds, China | Unicellular, epiphytic | *Zhang et al., 2019* |
| *Aphanocapsa sp.* Strain CCNUM3 | Humid moss on limestone in rain forest, China | Unicellular, epiphytic | *Zhang et al., 2019* |
| *Chrooccidiopsis spp.* Strains CCNUC1, CCNUC2, CCNUC3 | Arid and humid moss on limestone in rain forest, China | Unicellular, epiphytic | *Zhang et al., 2019* |
| *Chrooccidiopsis sp.* Strain CCNUM1 | Biofilm on humid limestone in rain forest China | Unicellular, epilithic | *Zhang et al., 2019* |
| *Cf. Leptolyngbya sp.* Strain CCNUW1 | Freshwater macrophytes in rain forest ponds, China | Filamentous, epiphytic | *Zhang et al., 2019* |
| *Cf. Leptolyngbya sp.* Strain CCNUW2 | Humid moss on limestone in rain forest, China | Filamentous, epiphytic | *Zhang et al., 2019* |
