# Supplementary material for: Substantial near-infrared radiation-driven photosynthesis of chlorophyll f-containing cyanobacteria in a natural habitat
Source: eLife. 2020 Jan 21;9:e50871. doi: 10.7554/eLife.50871 (PMC6974357; doi:10.7554/eLife.50871)
Supplement: Supplementary file 2. [file elife-50871-supp2.docx]

| **List of most abundant OTUs related to oxygenic phototrophs in black beachrock and seawater ^a^** | | | | |  |  |
| --- | --- | --- | --- | --- | --- | --- |
| **OTU ^b^** | **Best hit ^c^** | **Top layer (0-2 mm)** | **Green layer (2-5 mm)** | **Seawater** | | |
| OTU_019 | *Calothrix scopulorum* NIES-268 (100.0%) | 23.4 | 0.2 | 0.0 | | |
| OTU_002 | *Rivularia* sp. PCC 7116 (99.5%) | 16.5 | 0.0 | 2.4 | | |
| OTU_005 | *Halomicronema hongdechloris* C2206 (98.0%) | 9.8 | 79.9 | 0.0 | | |
| OTU_013 | *Chroococcidiopsis* sp. CCMP1489 (98.0%) | 7.1 | 0.1 | 0.0 | | |
| OTU_010 | *Cyanothece* sp. ATCC 51142 (93.6%) | 6.8 | 0.2 | 0.0 | | |
| OTU_035 | *Gomphosphaeria aponina* SAG 52.96 (92.6%) | 4.5 | 0.0 | 0.0 | | |
| OTU_015 | *Spirulina subsalsa* IAM M-223 (92.8%) | 3.7 | 0.0 | 0.0 | | |
| OTU_073 | *Chroococcidiopsis* sp. QUCCCM26 (97.4%) | 2.9 | 0.1 | 0.0 | | |
| OTU_081 | *Chroococcidiopsis* sp. QUCCCM26 (98.5%) | 2.4 | 0.0 | 0.0 | | |
| OTU_033 | *Chroococcidiopsis* sp. CCMP3187 (99.0%) | 2.4 | 0.0 | 0.0 | | |
| OTU_060 | *Microcoleus* sp. DAI (94.4%) | 2.4 | 0.0 | 0.0 | | |
| OTU_179 | *Cyanothece* sp. HPC-11 (94.2%) | 1.9 | 0.0 | 0.0 | | |
| OTU_117 | *Chroococcidiopsis* sp. CCMP3185 (97.4%) | 1.7 | 0.0 | 0.0 | | |
| OTU_072 | *Chroococcidiopsis* sp. QUCCCM26 (98.5%) | 1.5 | 0.0 | 0.0 | | |
| OTU_095 | *Hyella patelloides* LEGE 07179 (96.2%) | 1.3 | 0.0 | 0.0 | | |
| OTU_080 | *Chroococcidiopsis* sp. CCMP3185 (96.9%) | 1.3 | 0.0 | 0.0 | | |
| OTU_063 | *Dermocarpella incrassata* PCC 7326 (96.9%) | 1.1 | 0.0 | 0.0 | | |
| OTU_003 | *Pseudocapsa* sp. Ryu8-6 (95.4%) | 0.0 | 10.2 | 0.0 | | |
| OTU_006 | *Nodosilinea* sp. LEGE 06001 (93.1%) | 0.0 | 6.9 | 0.0 | | |
| OTU_070 | *Cyanothece* sp. SKTU126 (94.4%) | 0.0 | 2.2 | 0.0 | | |
| Other cyanobacteria | - | 9.4 | 0.2 | 80.0 | | |
| Chloroplasts | - | 0 ^d^ | 0 ^d^ | 17.6 | | |

^a^ The OTU included constitute at least 1% of all cyanobacterial-like sequences in either the top layer or the green layer. The abundance is shown as percentage of total cyanobacterial-like sequences; ^b^ The OTU sequences have been submitted to GenBank with accession numbers XXXXXXXX-XXXXXXXX; ^c^ Best hit to a cultured and named organism in the NCBI non-redundant database. Percentage sequence identity is shown in parenthesis; ^d^ No chloroplasts sequences were found.
